# Seroprevalence of *Leptospira* spp. antibodies in humans and livestock at various human-animal interfaces in Jordan

**DOI:** 10.14202/vetworld.2025.1-7

**Published:** 2025-01-09

**Authors:** Zuhair Bani Ismail, Lea Widemann, Yasmin Daradkeh, Omar Al-Omari, Alaa Fahmawi, Mais Lakaideh, Belal Sha’fout, Haia Mellhem, Leen Al-Bayari, Saad Al-Omari, Hani Talafha, Zaidoun Hijazeen, Bilal Al-Omari, Jean DeMarco, William B. Karesh, Ehab A. Abu-Basha

**Affiliations:** 1 Jordan University of Science and Technology, Irbid, Jordan; 2 EcoHealth Alliance, 520 Eighth Avenue, Ste. 1200, New York, USA; 3 King Abdullah University Hospital, Irbid, Jordan; 4 Food and Agriculture Organization of the United Nations (FAO), Amman, Jordan

**Keywords:** emerging zoonotic disease, enzyme-linked immunosorbent assay, human-animal interface, leptospirosis, One Health, seroprevalence

## Abstract

**Background and Aim:**

Leptospirosis, caused by *Leptospira* spp., affects humans and animals, posing significant health and economic burdens. This multidisciplinary One Health study aimed to estimate the prevalence of *Leptospira* spp.-specific antibodies in humans and livestock at the human-animal interfaces in Jordan.

**Materials and Methods:**

Study sites were selected across Jordan based on previous research showing a high likelihood of zoonotic pathogen circulation. The study population comprised 500 individuals and 400 livestock (sheep, goats, cattle, and camels). Blood samples were collected from both humans and livestock at baseline and at follow-up after 1.5 years. Humans were followed longitudinally, whereas livestock were sampled opportunistically. The samples were tested for *Leptospira* spp.-specific Immunoglobulin G antibodies using a commercially available enzyme-linked immunosorbent assay kit. For humans, seroprevalence was calculated from baseline data, and incidence was calculated using follow-up data. In livestock, seroprevalence was calculated at baseline and follow-up.

**Results:**

At baseline, the seroprevalence of leptospirosis in humans was 4.0% (95% confidence interval [CI]: 2.5–6.1), with exposure detected across all five sites. The incidence rate of human leptospirosis was 1145 cases/100,000 person-years (95% CI: 455–2,321). In livestock, the baseline seroprevalence was 1.7% (95% CI: 0.79–3.7), which increased to 4.3% (95% CI: 2.6–6.9) at follow-up. *Leptospira* spp. antibodies were detected only in sheep and goats at both sampling times, with no cases found in camels or cattle.

**Conclusion:**

This study highlights the presence and dynamics of leptospirosis at the human-animal interface in Jordan, revealing a baseline human seroprevalence of 4.0% and an incidence rate of 1145 cases per 100,000 person-years. Among livestock, seroprevalence increased from 1.7% to 4.3% over the study period, with exposure limited to sheep and goats. These findings underscore the importance of targeted public health and veterinary interventions to address zoonotic leptospirosis in endemic areas.

## INTRODUCTION

Leptospirosis is a re-emerging zoonotic disease that poses a major threat to the health of humans and animals worldwide. The disease is caused by certain types of *Leptospira* spp. bacteria that affect humans, rodents, cows, camels, sheep, goats, and wild animals [1–6]. Transmission occurs through either direct contact with or environmental exposure to the urine of infected animals.

Leptospirosis is an important occupational zoonotic disease [3, 5, 6]. Globally, it contributes to an estimated annual mortality rate of approximately 0.84 deaths/100,000 people [7]. The disease has a biphasic clinical presentation, starting with the septicemic phase, followed by immune manifestations [7, 8]. The most severe form of the disease, known as Weil syndrome, causes multisystem damage, including vascular, hepatic, renal, pulmonary, and skeletal muscle injury [7, 9]. Risk factors associated with human leptospirosis include temperate rainy climates, presence of rodents, wastewater disposal, garbage disposal facilities, history of contact with rats, presence of at-risk pets, proximity to rivers, history of floods, history of tidal inundation, and vegetation [10, 11].

Leptospirosis significantly impacts the health and production of livestock, leading to high treatment costs, loss of milk production, abortion, repeat breeding, stillbirth, and retained placenta [1, 12]. Several risk factors for leptospirosis have been reported, including age, large herd size, co-grazing with infected animals, contaminated water sources, natural breeding practices, inadequate husbandry practices, purchasing replacement heifers from infected herds, presence of dogs and cats in pastures, rodent contact with animal feed, calf-rearing facilities in close proximity to adult cows, and the use of dirty drenching equipment [13–15].

Although leptospirosis is the most common zoonotic infection worldwide, significant knowledge gaps persist regarding the epidemiology of leptospirosis in the Middle East [16]. Previously published findings revealed an alarmingly high prevalence of leptospirosis among apparently healthy dairy cows, as well as in cows with a recent history of abortion [15]. Despite these findings in animals, only one published report exists on leptospirosis in humans in Jordan [14].

Therefore, this study aimed to estimate the prevalence of *Leptospira* spp. antibodies in both humans and animals at the human-animal interface in Jordan.

## MATERIALS AND METHODS

### Ethical approval and Informed consent

The study protocols were reviewed and approved by the Institutional Review Board (IRB) of Jordan University of Science and Technology (JUST) (Approval number 3/137/2021) and the Health Media Lab (HML) IRB in Washington, DC, USA (Approval number E02947, 23/4/2021) for research involving human subjects. For the animal subjects, the protocols were approved by the Institutional Animal Care and Use Committees at Tufts University, USA, and JUST, Jordan (Approval number 10/8/2020 and 2023-05, respectively). All study team members completed the Collaborative Institutional Training Initiative modules for human subject research and ethical animal research. Informed written consent was obtained from the participants and the animal owners before sample collection.

### Study period and location

**Figure 1:**
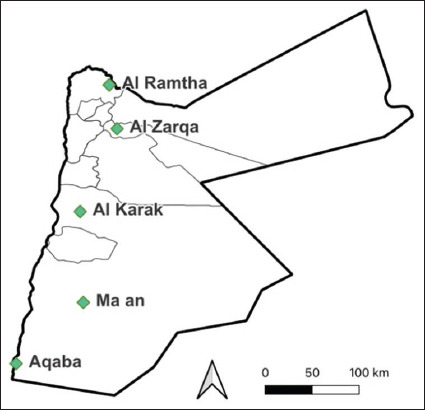
Study sites in Jordan where humans and livestock were sampled [The map was generated using ggplot2 version 3.5.1. 2016; Elegant Graphics for Data Analysis, USA].

The study was conducted from January 2022 to March 2024. The study sites were selected from five regions in Jordan: Al Ramtha, Al Zarqa, Al Karak, Ma’an, and Aqaba (Figure 1). The selection criteria were based on previous surveillance activities and the distribution of livestock. The sampling sites represented interfaces with camels, poultry, cattle, sheep, and goats, including multiple interface types. Using a randomized sampling generator in R Studio, one point per region was selected, and the three closest interface sites within 10 km of each point were selected. If necessary, the grid was expanded until all sites were found. The sites were visited randomly until all interface types and enrolment goals were met.

### Study population

The eligibility criteria included adults (18+) who could provide written informed consent and children (12+) who could give verbal assent with parental consent. Participants were allowed to withdraw at any time. A prescreening checklist was used to determine exposure status, which was defined as regular (monthly or more frequent) interaction with livestock or poultry.

The livestock studied included cattle, sheep, goats, and camels owned by participants in the selected regions. Animals underwent a brief physical examination to assess age, gender, recent pregnancy, and overall health, including body condition and signs of abnormalities, such as nasal discharge or diarrhea. In Jordan, vaccination against leptospirosis is not routinely practiced.

### Sample size calculation

The One Health parent study determined that a minimum of 200 unexposed and 200 exposed human participants were required. Exposure was defined as regular interactions with poultry and livestock. Based on 5% Type I error rate and 80% statistical power, this calculation estimated that 1% of unexposed and 12.5% of exposed individuals would test positive for one of four zoonotic diseases [17]. Based on the Fleiss method with continuity correction, the sample size was 400 participants. To account for 20% loss to follow-up, the sample size was adjusted to 500. The participants were evenly distributed across five regions, with each region aiming for 50 unexposed and 50 exposed individuals.

The sample size for livestock was determined to be 385 based on a 95% confidence level, a margin of error of ±5%, and a population proportion of 50%. To ensure robust data collection, 400 animals were sampled, comprising 20 from each taxon (camels, sheep, cattle, and goats) per site per visit (total of 5 visits).

### Sample collection

Blood samples were collected from the participants at baseline and follow-up. Approximately 5–8 mL of whole blood was drawn from each participant using vacutainer needles and plain clotting tubes.

For livestock, blood samples were also collected at baseline and follow-up. About 5–8 mL of whole blood was obtained from cattle, camels, sheep, and goats through jugular venipuncture using vacutainer needles and plain clotting tubes.

Samples were immediately placed in an icebox containing ice packs and transported to the laboratory, with travel times ranging from 2 to 24 h depending on the site location. In the laboratory, samples were centrifuged at 10,000× *g* for 10 min to collect serum. The serum was stored at –70°C to –80°C until analysis.

### Laboratory testing

Serum samples were analyzed using a comme-rcially available enzyme-linked immunosorbent assay kit (ELISA) (Sincere Biotech, Beijing, China) with a multispecies conjugate to detect antibodies against *Leptospira* spp. The ELISA test had a sensitivity of 100% and specificity of 96%. The intra-assay and inter-assay coefficients of variation were <10% and 15%, respectively.

### Statistical analysis

Laboratory results and demographic data for humans and animals were entered into Airtable and exported to R software version 4.3.2 (R Core Team, 2023) for statistical analysis. Seroprevalence was estimated as the proportion of seropositive cases per sampling round, with 95% confidence intervals (CI) reported. For animals, seroprevalence was calculated separately for each sampling round due to potential re-sampling at follow-up. For humans, seroprevalence was calculated only at baseline, with follow-up data used to determine the incidence among those initially disease-free. Incid-ence was defined as negative at baseline and positive at follow-up. Incidence was calculated using a Poisson model with an offset for varying follow-up durations, and the rate per 1000 person-years was derived from the model’s intercept term, with 95% CI obtained using the profile likelihood method.

## RESULTS

A total of 500 participants were enrolled at baseline (Table 1). The majority of participants were male (78%) and aged 18–49 (78%). The baseline seroprevalence was 4% (95% CI: 2.5–6.1), with no significant regional differences (Table 2). The highest seroprevalence was observed in Al-Ramtha (6%), followed by Ma’an (5%), Al-Zarqa (4%), Aqaba (3%), and Al-Karak (2%). There were no significant differences in seroprevalence between exposed (2.85%, 95% CI: 1.15–5.77) and unexposed (5.12%, 95% CI: 2.79–8.69) individuals. The incidence of human leptospirosis was 1145 cases/100,000 people/year (95% CI: 455–2321).

**Table 1:** Demographic characteristics of human participants at baseline.

Variable	n = 498	Percentage
Livestock exposure		
Exposed	202	41
Unexposed	296	59
Gender		
Female	110	22
Male	338	78
Age		
<18	40	8.0
18–29	180	36
30–49	208	42
50+	70	14
Monthly income		
250–500 JD	484	97
501–1000 JD	13	2.8
Highest education		
None	35	7.0
Primary	95	19
Secondary	272	55
University	95	19

**Table 2:** Number and percentages of human samples positive for *Leptospira* spp. antibodies by site at baseline.

Site	Tested (n = 500^1^)	Positive (n = 20^1^)
Al-Ramtha	100	2 (2)
Al-Karak	100	6 (6)
Ma’an	100	4 (4)
Aqaba	100	3 (3)
Al-Zarqa	100	5 (5)

1 n (%)

A total of 700 animals were sampled (Table 3), including 200 camels, 200 goats, 200 sheep, and 100 cows. Most camel herds were small (1–25 animals), while herds of cattle, sheep, and goats were larger (over 25 animals). The majority of sampled animals were female and aged under 5 years.

**Table 3:** Characteristics of livestock sampled at baseline and follow-up.

Variable	Sheep		Goat		Cattle		Camel	
			
	Baseline (n = 100)	Follow-up (n = 100)	Baseline (n = 100)	Follow-up (n = 100)	Baseline (n = 50)	Follow-up (n = 50)	Baseline (n = 100)	Follow-up (n = 100)
Site								
Al-Karak	20	20	20	20	10	0	20	20
Al-Ramtha	20	20	20	20	20	20	20	20
Al-Zarqa	20	20	20	20	15	20	20	20
Aqaba	20	20	20	20	0	0	20	20
Ma’an	20	20	20	20	5	10	20	20
Herd size								
1–25	27	30	27	30	5	10	78	68
25–100	45	56	45	56	45	40	22	12
100–300	13	14	13	14	0	0	0	5
300+	15	0	15	0	0	0	0	15
Age								
<6 months	9	11	9	11	24	34	24	21
6 month. - <1 year	14	16	14	16	1	7	5	7
1–5 years	50	66	50	66	21	8	40	41
5+ years	27	7	27	7	4	0	31	31
Sex								
Female	87	88	87	88	31	28	74	61
Male	13	12	13	12	19	22	26	39

At baseline, the overall seroprevalence of lepto-spirosis in livestock was 1.7% (95% CI: 0.79–3.7) (Table 4). The highest positivity rate was observed in Al-Ramtha, with 4/80 samples (5%) testing positive. Aqaba and Al-Zarqa had lower positivity rates of 1.7% (1/60 samples) and 1.3% (1/75 samples), respectively. No positive samples were detected in Al-Karak and Ma’an.

**Table 4:** Number and percentage of livestock samples positive for *Leptospira* spp. antibodies by site at baseline.

Site	Tested (n = 350^1^)	Positive (n = 6^1^)
Al-Ramtha	80	4 (5)
Al-Karak	70	0 (0)
Ma’an	65	0 (0)
Aqaba	60	1 (1.7)
Al-Zarqa	75	1 (1.3)

1 n (%)

**Figure 2:**
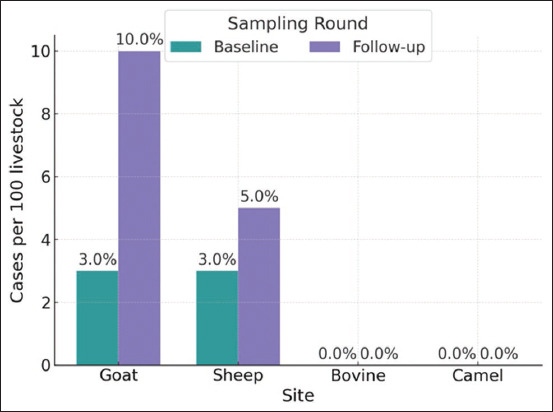
Percentage of *Leptospira* spp. antibody-positive samples by livestock species.

At follow-up, the overall seroprevalence of lepto-spirosis in livestock was 4.3% (95% CI: 2.6–6.9) (Table 5). Ma’an showed the highest positivity rate at follow-up with 7.1% (5/70 samples), followed by Al-Karak with 6.7% (4/60 samples), Aqaba with 5% (3/60 samples), and Al-Ramtha with 3.8% (3/80 samples). Al-Zarqa had no positive samples at follow-up. At baseline, both goats and sheep had a seroprevalence of 3% for *Leptospira* spp. antibodies (Figure 2). By follow-up, the rate increased to 10% in goats and 5% in sheep. No cases of cattle or camels were detected during either visit.

**Table 5:** Number and percentages of livestock samples positive for *Leptospira* spp. antibodies at follow-up.

Site	Tested (n=350^1^)	Positive (n=15^1^)
Al-Ramtha	80	3 (3.8)
Al-Karak	60	4 (6.7)
Ma’an	70	5 (7.1)
Aqaba	60	3 (5)
Al-Zarqa	80	0 (0)

1 n (%)

## DISCUSSION

This study is the first in Jordan to use the One Health approach to investigate the seroprevalence of *Leptospira* spp. antibodies in both humans and animals, focusing on the human-animal interface. Our findings indicate a seroprevalence of 4% for leptospirosis in humans, which is consistent with 3.3% estimated by Obaidat *et al*. [14] in a large cross-sectional study across 11 Jordanian governorates. Globally, leptospirosis poses a significant health burden, accounting for over 1 million cases and nearly 60,000 deaths annually in humans [16]. Due to their non-specific symptoms and limited diagnostic capacity, many cases are likely unrecognized or unreported [15, 18–22]. The seroprevalence of leptospirosis in the Middle East is generally scarce. Yemen shows high seroprevalence rates of 41.3% among slaughterhouse workers [19], whereas Iran reported a much lower rate of 1.1% [20]. These discrepancies can be attributed to various factors, including environmental conditions, socioeconomic status, and healthcare availability. Yemen’s higher prevalence is possibly due to its tropical climate, inadequate healthcare, and poor sanitation, whereas Iran benefits from drier climates and better healthcare infrastructure. In Jordan, with seroprevalence rates intermediate between Yemen and Iran, factors such as regional climate, socioeconomic conditions, and healthcare resources are likely to influence observed rates.

In this study, the seroprevalence of leptospirosis was 2.85% in the exposed group (95% CI: 1.15–5.77), compared to a higher rate of 5.12% (95% CI: 2.79–8.69) in the unexposed group. These results suggest several important considerations: Typically, one would expect higher seroprevalence in the exposed group due to greater potential contact with sources of infection. However, the higher seroprevalence observed in the unexposed group contradicts this expectation. The CIs for both groups were relatively wide and overlapped slightly, indicating that the difference in seroprevalence between the exposed and unexposed groups was not statistically significant. Possible explanations for these findings include misclassification of exposure status, higher overall exposure risk in the environments of the unexposed group, such as other reservoir species not studied here, and more effective protective behaviors among the exposed group. These results highlight the complexity of leptospirosis transmission dynamics and suggest that public health interventions should address a wide range of environmental and community-level risk factors rather than focusing solely on high-risk occupations or known exposures. Further research is needed to investigate the factors contributing to the higher seroprevalence in the unexposed group, including environmental and behavioral variables, as well as potential misclassification, to guide more effective prevention and control strategies.

This study indicates that the incidence of human leptospirosis is significantly high at 1145 ca- ses/100,000 people/year (95% CI: 455–2321) compared with many documented outbreaks in tropical and subtropical regions, where such incidence rates often reach 100/100,000 inhabitants/year [23]. This exceptionally high incidence rate underscores the significant public health burden of leptospirosis in the studied area regardless of exposure to livestock, highlighting the urgent need for intensified surveillance, targeted interventions, and further research to identify and address the factors contributing to such elevated incidence levels.

Results from this study revealed that at baseline, both goats and sheep exhibited a seroprevalence of 3% for *Leptospira* spp. antibodies. By follow-up, this seroprevalence increased to 10% in goats and 5% in sheep. No positive cases were found in cattle or camels at either time point. The consistent presence of *Leptospira* spp. antibodies in goats and sheep, along with the absence of cases in cattle and camels, indicates that goats and sheep might be more frequently exposed to *Leptospira* spp. and could play a more critical role in local leptospirosis transmission.

In contrast, a previous study by Ismail *et al*. [4] in Jordan has reported a much higher seroprevalence of 26.25% in dairy cattle. In addition, a study from the United Arab Emirates reported seroprevalence rates of 4.1% in camels and 1.7% in cattle [23], whereas a study from Saudi Arabia reported a 6.7% prevalence in camels [24]. The observed discrepancies in seroprevalence may be attributed to differences in study populations, sampling methods, and regional variations. Seasonal factors, livestock management practices, and regional climate, such as rainfall and temperature, may also influence the prevalence and detection of *Leptospira* spp. Moreover, species susceptibility and immune response variations could contribute to these disparities, with some species potentially showing higher resistance [25]. Further research is required to explore these factors and better understand the transmission dynamics of *Leptospira* in livestock.

## CONCLUSION

This study highlights the critical role of the One Health approach in understanding zoonotic diseases, focusing on the seroprevalence of *Leptospira* spp. antibodies in humans and livestock at the human-animal interface in Jordan. The findings reveal a baseline human seroprevalence of 4% and a significant increase in livestock seroprevalence from 1.7% to 4.3% over the study period, particularly in sheep and goats, while cattle and camels showed no detectable antibodies. These results suggest that sheep and goats may serve as key reservoirs for leptospirosis transmission in the region. Furthermore, the absence of a strong correlation between livestock exposure and human infection underscores the potential importance of environmental factors, such as contaminated water or soil, in the disease’s transmission dynamics.

The practical implications of this research are substantial, providing a foundation for enhanced public health policies, improved livestock management practices, and targeted environmental interventions. By addressing gaps in diagnostic capacity and expanding surveillance efforts, stakeholders can better mitigate the risks of leptospirosis. Future research should focus on refining diagnostic tools, exploring alternative transmission pathways, and investigating region-specific environmental and socio-economic factors influencing disease spread. This integrated approach can inform effective prevention and control measures, ultimately reducing the burden of leptospirosis on human and animal populations alike.

## AUTHORS’ CONTRIBUTIONS

ZBI and LW: Interpreted the results, statistical analysis, and drafted, reviewed and edited the manuscript. YD, ZH, LAB, and HT: Collected the animal samples. OAO, ML, BS, SAO: and HM: Collected human samples. JD: Project management. BAO and AF: Conducted laboratory analysis and data curation. WBK: Conceptualization and project management. EAAB: Supervised field and laboratory work, project administration, conceptualization, and funding acquisition. All authors have read and approved the final manuscript.
